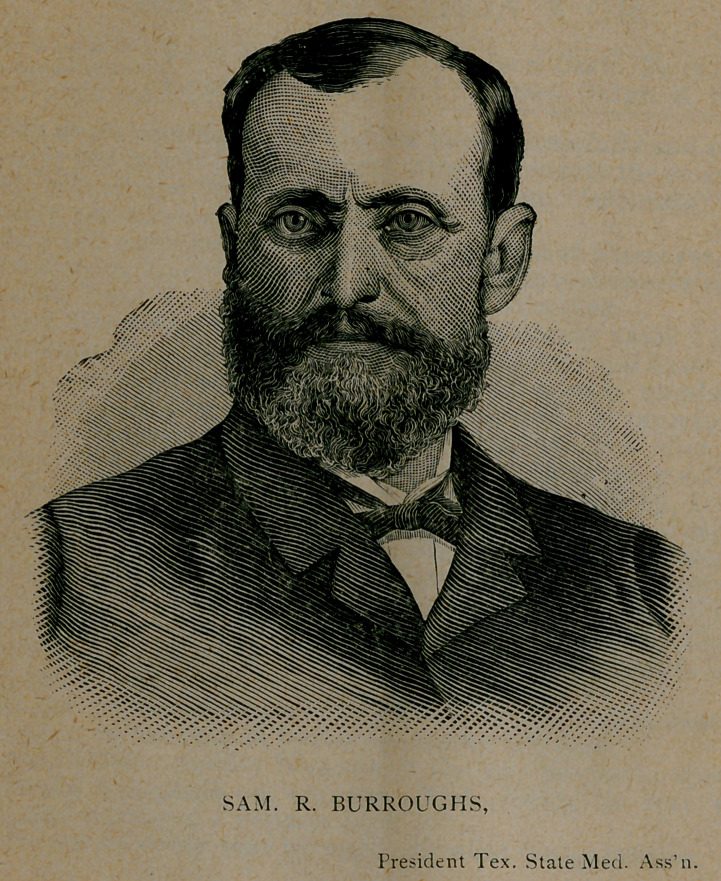# The President Elect, Texas State Medical Association, Dr. Sam. R. Burroughs

**Published:** 1887-05

**Authors:** 


SAM. R. BURROUGHS,

President Tex. State Med. Ass'n.

### Editorial Department.

F. E. DANIEL, M. D., EDITOR AND PUBLISHER.

COLLABORATORS:

*E. J. Doering, M. D., Chicago.*

*Geo. Cupples, M. D., San Antonio.*

*Odo Belz, M. D., Germany.*

*E. J. Beall, M. D., Fort Worth, Texas.*

*T. C. Osborn, M. D., Cleburne, Texas.*

*Wm. Penny, M. D., New York.*

*H. O. Marcy, M. D., Boston.*

*C. K. Gregg, M. D., Mexico.*

*R. M. Swearingen. M. D., Austin.*

*C. H. Wilkinson, M. D., Galveston.*

*J. W. McLaughlin, M. D., Austin.*

*R. H. L. Bibb, M. D., Mexico.*

### THE PRESIDENT ELECT T. S. M. A.

### DR. SAM R. BURROUGHS OF LEON COUNTY.

Dr. Samuel Raymond Burroughs of Leon County, Texas, upon whom the State Medical Association has just conferred the highest honor within its gift, the Presidency of the Association, was born in Tuscaloosa County, Alabama, October 3, 1842, and is therefore now in his forty-fifth year. He is of Scotch-Irish descent, and is a son of Benjamin F. and Louisa Fair Burroughs of Alabama.

Dr. Burroughs received a thorough English education at Palestine (Texas) High School, and at Mt. Prairie Institute, Anderson County, Texas, and in 1858, begun the study of medicine at Palestine, under the instruction of Dr. T. N. Rhoads and Dr. W. S. A. Kirksey. He attended two courses of lectures, 1866–7, at Galveston, in the Medical Department of the Texas State University, and graduated from that Institution in 1869, and afterwards, in 1873, from the Galveston Medical College and Hospital. Shortly afterwards he was elected by the Concours Board of Examiners to the Chair of Chemistry and Toxicology in the latter school, which position he held two years, and resigned in '78 on account of bad health. He was also made Dean of the Faculty, and was a member of the Board of Trustees.

Dr. Burroughs. after receiving his first diploma, settled in Leon County and engaged in practice, (1867 to 1876, except the interval of his connection with the college); thence removed to Houston, where he practiced two years; but on account of ill health he returned to Leon County, where he resides at the present time (Raymond P. O). He was the President of Leon County Medical Society two consecutive years, and was very active in promoting organization in other counties. He was also, under the law of 1873, a member of the Leon County Board of Medical Examiners, and has since 1876, been an active and zealous member of the Texas State Medical Association. As Chairman of the Committee on Collective Investigation of Disease, a position he has held two consecutive terms Dr. Burroughs devoted much time and study to the subject and managed to awaken an interest in it on the part of the profession not felt before; and his reports in the Transactions of the State Association, reflect credit upon him and upon the profession.

During the war Dr. Burroughs served as private in Company G., 1st Texas Volunteers, Hood's Brigade; and at the battle of Chicamauga was taken prisoner. He was sent to Camp Douglass prison in Illinois, where he was confined till the close of the war, serving his fellow captives efficiently as Prison Hospital Druggist.

Surgery and gynæcology have claimed most of the Doctor's attention, though he has done a general practice. Of his many valuable contributions to medical literature, may be mentioned: "Spurious Melanosis," (Galveston Medical Record, '68); Hæmaturia Miasmatica," (Texas Medical Journal, 1874); "The Hymen; its Anatomy, Malformations and Malpositions," (Trans Texas State Medical Association, 1876); "Report on Medical Resources of Texas," (as Chairman of Committee, Trans Texas State Medical Association, 1877); "What are the Post-Mortem Evidences of Virginity as Determined by Examination of the Uterus and its Appendages, excluding the External Organs of Generation"? (Trans T. S. M. A., 1879, and Tex. Med. and Surg. Record, 1879); "Report on Chemistry," as Chairman of Section (Trans T. S. M. A., 1883); "Report on Collective Investigation of Disease." as Chairman of Committee (Trans T. S. M. A., 1885–6–7.)

A valuable and useful instrument—"a double current tube, with outer attachment and stop cocks, for washing out cavities in Empyæmia," is the invention of this gentleman, who has used it; but it seems never to have been extensively made known to, or used by the profession, owing doubtless to the Doctor's well known and characteristic modesty.

Dr. Burrough3 is one of the working men of the association, and rarely fails to attend its meetings. His interest amounts almost to an enthusiasm, and his popularity is universal. In this instance evidently, the office sought the man, and the election was a spontaneous tribute to his merit, which all recognize. He is of the intellectual type, and modesty is one of his chief characteristics. Calm, dignified and self-possessed, and with a good knowledge of parliamentary law, supported, as he is, by a consciousness of the sympathy and respect of the entire body, Dr. Burroughs will doubtless make a most excellent president and will shed additional honor on the association. We produce herewith a fair portrait, from which it may be seen that intelligence and firmness are blended with his many other virtues. Dr. Burroughs is a happy husband and father, and lives in a quiet, cosy home in Leon county, in the heart of a rich agricultural country. His wife was Rebecca A. Henry of Pickens County, Alabama; they were married in '67, and have four children—three girls and one son; the eldest now 18 years of age.

We present our distinguished President as a splendid type of the Texas country physician—a class to whom the world owes much—from whom originate, it is admitted, most of the useful discoveries and inventions in medicine—and we are proud of him!

## Figures and Tables

**Figure f1:**